# Needle Tract Seeding of Thyroid Follicular Carcinoma after Fine-Needle Aspiration

**DOI:** 10.1155/2020/7234864

**Published:** 2020-02-22

**Authors:** Yuxin Guo, Adrian Jit Hin Koh

**Affiliations:** Department of General Surgery, Changi General Hospital, Singapore

## Abstract

*Introduction*. Fine-needle aspiration (FNA) biopsies are fundamental procedures in the diagnoses of thyroid tumours. Reports of needle tract tumour seeding, however, question its practice regarding patient safety and potentially jeopardizing its widespread usage. *Case Report*. We describe a case of a 50-year-old lady with known multinodular goitre, and previous fine-needle aspiration (FNA) biopsies of her thyroid nodules in 2010, who developed palpable right neck nodules 8 years after the initial FNA. Imaging and histological biopsies revealed suspicious right sternocleidomastoid (SCM) nodules that are likely needle tract tumour deposits. She underwent a total thyroidectomy with central compartment clearance and excision of the right SCM nodules and received radioactive iodine therapy thereafter. *Discussion*. Contrary to other forms of malignancies, needle tract seeding is an uncommon occurrence for thyroid cancers. Nevertheless, there is speculation regarding its potential in cutaneous spread of malignancy with studies investigating its optimal techniques and application. *Conclusion*. While FNA remains an indisputable tool in the management of thyroid tumours, precautions must be taken to safeguard patient safety and improve patient outcomes.

## 1. Introduction

Fine-needle aspiration (FNA) refers to the diagnostic technique of obtaining cellular material in establishing a cytological or pathological diagnosis, and the use of FNA has become common place in the management of thyroid nodules and its disorders. While tumour seeding from FNA is considerably lower for thyroid cancers relative to other malignancies, there are nonetheless reports of thyroid needle tract tumour seeding which raises concerns regarding its widespread usage. The utility of FNA as a quick and cost-effective tool is, however, undeniable with its application and interpretation refined from its initial appraisal in 1993 by Gharib and Goellner [1]. As such, we report a case of needle tract seeding of thyroid follicular carcinoma and reviewed existing literature of such occurrences.

## 2. Case Report

A 51-year-old lady with invasive thyroid follicular carcinoma presented with needle tract deposits in her right sternocleidomastoid (SCM) and platysma muscle. Her medical history includes hypertension and a multinodular goitre, for which she underwent FNA in 2010, of the dominant 1.8 cm solid right thyroid lower pole nodule with a calcified rim. The procedure was performed under ultrasound guidance with 4 sampling passes using a 23-gauge needle. Histology showed suspicions of a follicular neoplasm for which the patient was offered surgical resection but declined and defaulted follow-up thereafter.

**Figure 1:**
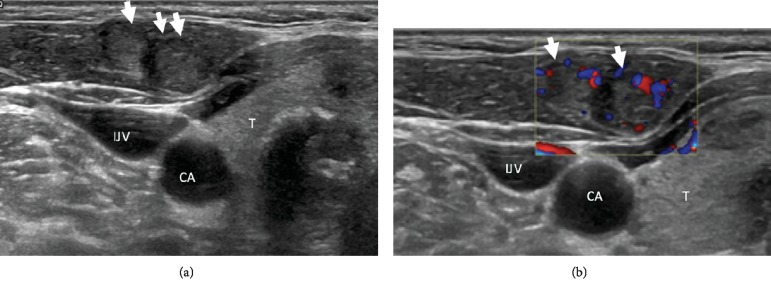
Arrows point the 2 nodules within the right sternocleidomastoid muscle on ultrasound. IJV, internal jugular vein; CA, carotid artery; T, thyroid.

**Figure 2:**
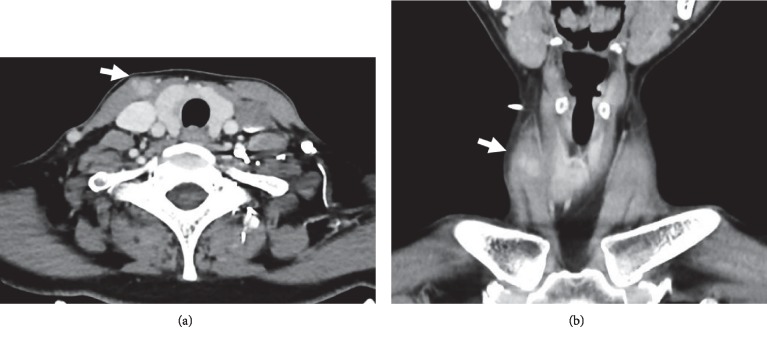
Right sternocleidomastoid muscle nodules demonstrated by white arrows on computed tomography of the neck in (a) axial view and (b) coronal view.

She returned in August 2018 with a new right lower neck nodule. On examination, the right thyroid lower lobe nodule was palpable measuring 2 cm, along with a superficial right lower neck 0.5 cm nodule. An ultrasound thyroid demonstrated multiple stable thyroid nodules with the dominant right lower pole nodule measuring 1.8 × 1.4 × 1.2 cm, an indeterminate 0.7 cm right lower neck subcutaneous nodule, and 2 new heterogenous nodules with internal vascularity within the right SCM at 1.1 × 0.8 × 0.7 cm and 0.9 × 0.6 × 0.5 cm (Figure 1). FNA reiterated findings of a follicular neoplasm for the right lower pole nodule with suggestions of thyroid follicular lesions for the right lower neck subcutaneous nodule and right SCM nodules with no lymphoid yield identified. Computed tomography (CT) neck was also performed, illustrating findings congruent to the thyroid ultrasound with no regional lymphadenopathy (Figure 2).

**Figure 3:**
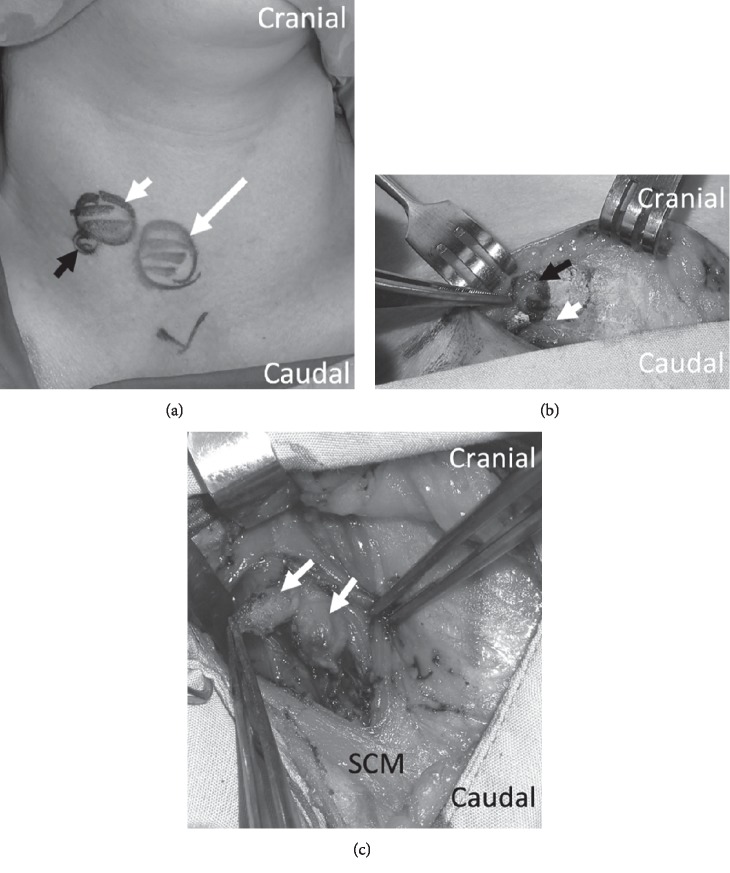
Intraoperative pictures: (a) skin markings prior to incision, right thyroid nodule (long white arrow), right SCM nodules (short white arrow), platysma nodule (black arrow), (b) right platysma nodule (black arrow) with SCM underneath (white arrow), and (c) 2 right SCM nodules (white arrows). SCM, sternocleidomastoid muscle.

The patient subsequently underwent a total thyroidectomy, right central compartment clearance, excision of right SCM and platysma nodules with reimplantation of the bilateral parathyroid glands on 27^th^ August 2018. Intraoperatively, there was a right platysma nodule with 2 right SCM nodules along the same tract, consistent with possible FNA tract seeding (Figure 3). Postoperative recovery was uneventful, and the patient was discharged well and stable on postoperative day 4.

Histology of the resected specimen confirmed a widely invasive follicular carcinoma of the right thyroid lobe invading the isthmus and extrathyroidal tissue, measuring 3 cm in maximum dimension, with capsular and vascular invasion. The right SCM and platysma nodules were positive for deposits of follicular carcinoma. Meanwhile, none of the 11 lymph nodes harvested showed evidence of metastatic carcinoma, and resection margins were clear of malignancy. Final histology report revealed a T3bN0MX right thyroid follicular carcinoma with needle tract deposits in the right SCM and platysma. Discussions at a head and neck oncologic multidisciplinary meeting established the likelihood of needle tract deposits on a background of invasive follicular carcinoma of the right thyroid lobe and recommended for postoperative radioactive iodine therapy (RAI). Following RAI, an I-131 whole body scan with single-photon emission CT (SPECT-CT) of the neck and thorax was performed on 5^th^ October 2018, revealing uptake in multiple small bilateral lung nodules suspicious for metastases with no other suspicious uptake. The patient remained well and stable upon review in February 2019 with no evidence of tumour recurrence.

## 3. Discussion

Unlike other tumours including hepatocellular carcinoma and pancreatic tumours, thyroid FNAs are deemed to be at lower risk for needle tract seeding of neoplastic cells [2–4]. Reports of thyroid FNA tumour seeding are few and far between with their techniques refined over the years in minimising such postulated risks.

FNA is a vital tool in the diagnosis and management of thyroid tumours. While exceedingly rare, there are concerns regarding its potential for tumour seeding. Tumour cells can be disseminated along the puncture tract when the aspirate needle is withdrawn [5]. These cells can then propagate, forming distant islands or “seeds” from the original tumour. In our patient's case, the right platysma and SCM nodules are likely the cause of direct needle tract tumour seeding, which occurred 8 years from her previous FNA in 2010. Any suggestion of distant metastases to these sites would be peculiar given that (1) these are uncommon sites of distant tumour spread from thyroid neoplasms and (2) the right platysma and SCM nodules developed along a straight line that can be traced from the skin to the thyroid nodule, suggesting seeding along the tract of previous needle puncture for FNA.

Various technical recommendations to minimise the risks of tumour seeding, including the usage of ultrasound guidance, an experienced operator, smaller gauge needles, fewer sampling passes, and the release of negative pressure during needle withdrawal, have been reported [6–8]. The use of smaller gauge needles, in particular, has been an area of contention. Cases of needle tract seeding occurred predominantly in cases where needles of 23 gauge or larger were used [7]. There is no clear consensus regarding optimal needle gauge for FNA with some recommending 21–27-gauge needles [1, 6, 9, 10] and others advising smaller 25–27-gauge needles, citing no differences in diagnostic yield [8, 11, 12]. These recommendations, however, remain postulations for risk-reduction, due to the infrequency of thyroid FNA needle tract seeding. Taking into account the countless thyroid FNAs that are performed, the application of these techniques may prove counterproductive and has to be balanced with the need for sufficient specimen yield for assessment.

Besides technical circumstances, tumour “aggression” and propensity for dissemination may also contribute to the risks of needle tract seeding. A review of the existing literature revealed 3 other cases of cutaneous dissemination of follicular thyroid cancers with time from FNA-to-seeding ranging between 1 month and 5 years [4, 13, 14]. While the time to the presentation of cutaneous dissemination varied significantly, 2 cases with earlier presentations reported by Panunzi et al. [13] and Ito et al. [15] exhibited features predisposing to a biologically more aggressive tumour. The former was afflicted with multiple myeloma and, having undergone chemotherapy, is significantly immunodeficient, thus posing a risk in propagating neoplastic cells [16]. Meanwhile, the patient reported by Ito et al. had cells with high proliferative activity with the presence of biological markers Ki-67 and galectin-3, both with possible roles in the development of follicular carcinoma [15]. Meanwhile, our case demonstrated an aggressive tumour with capsular and vascular invasion, possibly contributing to the development of tumour “seeds” within the right SCM and platysma even after 6 years from initial FNA. The prolonged duration to discovery of tumour seeding may have contributed to heightened disease dissemination resulting in multiple lung metastases. In contrast, the patient in Uchida et al.'s report was fairly quiescent, showing invasion through tumour capsule with no other features reflecting tumour aggression [14]. Even with the resurfacing of needle tract tumour deposits after 5 years from FNA, there was no evidence of disease progression or dissemination.

## 4. Conclusion

Thyroid FNA remains an indispensable tool in the guidance of thyroid nodule management. While needle tract seeding is uncommon, various technical precautions may be undertaken to reduce such occurrences, thereby safeguarding patient safety while ensuring precise diagnosis and treatment.
